# Efficient removal of bovine serum albumin from water by cellulose acetate membranes modified with clay and titania nano particles

**DOI:** 10.3389/fchem.2023.1111558

**Published:** 2023-02-01

**Authors:** Heba M. Refaat, Nada Ashraf, Ali El-Dissouky, Hossam A. Tieama, Elbadawy A. Kamoun, M. S. Showman

**Affiliations:** ^1^ Department of Chemistry, Faculty of Science, Alexandria University, Alexandria, Egypt; ^2^ Abu Qir Fertilizers and Chemical Industries Co., Alexandria, Egypt; ^3^ Nanotechnology Research Center (NTRC), The British University in Egypt, Cairo, Egypt; ^4^ Department of Polymeric Materials Research, Advanced Technology and New Materials Research Institute, City of Scientific Research and Technological Applications, Alexandria, Egypt; ^5^ Department of Fabrication technology, Advanced Technology and New Materials Research Institute, City of Scientific Research and Technological Applications, Alexandria, Egypt

**Keywords:** ultrafiltration, nano-clay, nano-titanium oxide, bovine serum albumin, water remediation

## Abstract

Modified cellulose acetate membranes with bentonite clay (CA/bent) and TiO_2_ nanoparticles (CA/TiO_2_) using the phase inversion method are successfully prepared and characterized. These Membranes are favored due to their high salt rejection properties and recyclability. The IR and EDX spectral data indicate the formation of modified membranes. The Scan Electron Microscope micrographs show that the modified membranes have smaller particle sizes with higher porosity than the neat membrane. The average pore diameter is 0.31 µm for neat cellulose acetate membrane (CA) and decreases to 0.1 µm for CA/0.05bent. All modified membranes exhibit tensile strengths and elongation percentages more than the neat membrane. The higher tensile strength and the maximum elongation% are 15.3 N/cm^2^ and 11.78%, respectively, for CA/0.05bent. The thermogravimetric analysis of modified membranes shows higher thermal stability than the neat membrane. The modified membranes exhibit enhanced wettability and hydrophilicity compared with cellulose acetate, by measuring the contact angle which decreases from 60° (CA) to 40° (CA/0.1bent). The ultrafiltration tests indicated that the CA/bent and CA/TiO_2_ are better than CA. The most efficient nanocomposite membrane is CA/0.05bent with 100% removal of (BSA) from industrial water with a flux equal to 9.5 mL/min under an applied pressure of 20 bar. Thus, this study introduces a novel ultrafiltration membrane (CA/0.05bent) that can be used effectively to completely remove bovine serum albumin from contaminated water.

## 1 Introduction

Water deficiency and clean drinking water demand have great attention nowadays. So, it is essential to develop new suitable methodologies for water treatment. Membranes are favored over many other technologies for water treatment of saline and wastewater, desalination of seawater sewage treatment, and other applications, as they don`t need chemical additives due to their low energy consumption, low cost, and eco-friendly (Akram et al., 2018; Divya and Oh, 2022). Membranes are categorized into polymeric and inorganic membranes. Inorganic membranes are made up of metals or ceramics with high contractual, mechanical, and thermal strengths. Even though they have extremely high discernment, their low permeability makes them not substantial for several applications (Joshi et al., 2015; Iftikhar et al., 2021; Iftikhar et al., 2022). Polymeric materials can be used instead of inorganic membranes (Ma et al., 2012) due to their greater flexibility, ease of production, chemical stability, and mechanical stability. Also, their pore sizes are more suitable for various filtration processes such as reverse osmosis (RO), ultrafiltration (UF), nanofiltration (NF), and microfiltration (MF) (Yampolskii, 2012). They show weaknesses such as relative of high energy, low permeability, short lifetime, and low resistance to fouling. Membrane fouling is a severe problem for membrane materials used in all the previous pressure-driven processes. So, it is vital to improve low-energy, economically sound, and well-designed membranes for removing all water pollution. Traditional methods involved material cleaning by backwash and chemical cleaning using acids, hypochlorite, and sodium hydroxide, but it is an ineffective approach as it may cause membrane damage (Türkoğlu Demirkol et al., 2017).

Recently membrane surfaces were modified by different chemical additives; organic, inorganic, hybrid, and biomaterials such as chitosan, starch, activated carbon, siloxane, alumina, graphene oxide, mesoporous silica (SiO_2_), titanium oxide (TiO_2_) (Wang et al., 2021), and zeolite (Li et al., 2016). They show weaknesses such as relative consumption of high energy, permeability, dumpy lifetime, and low resistance to fouling. Membrane fouling is a severe problem for membrane materials used in all the previous pressure-driven processes. So it is vital to improve low-energy, economical, and well-designed membranes for removing all water pollution. Traditional methods involved material cleaning by backwash and chemical cleaning using acids, hypochlorite, and sodium hydroxide, but it is a disabled approach as it may cause membrane damage (Türkoğlu Demirkol et al., 2017).

Additives increase pore numbers and pore area, change the membrane’s hydrophilicity, and improve water permeability-increasing water flux and preventing swelling (Yuan and Dan-Li, 2008; Saljoughi et al., 2009; Simone et al., 2010; Zhang et al., 2011; Jiang et al., 2013; Vatsha et al., 2013; Lee et al., 2015; Peydayesh et al., 2017). They can be used as a single ingredient or as a mixture of different components (Peeva et al., 2012).

Due to its photocatalytic and hydrophilic characteristics, nano-titanium dioxide (TiO_2_) has developed as an extraordinary material for the creation of nano-composite membranes for breaking down organic toxins in wastewater treatment. Because of its antifouling ability, hydrophilicity, and high stability, TiO_2_ nanomaterial is commonly active in membrane manufacturing (Alzahrani and Mohammad, 2014; Zhao et al., 2015). Also, Clays have attracted special attention among the numerous inorganic fillers because of their compatible distribution in the polymer matrix, high surface area, economic, non-toxicity, and natural accessibility (Alekseeva et al., 2019a; Alekseeva et al., 2019b). In our work cellulose acetate, ultrafiltration membrane was prepared and modified with nanoTiO_2_ and nano-clay, to enhance protein removal from drug and food industry wastewater. Studying their mechanical and thermal stability, wettability, ion exchange capacity, flux, and BSA rejection will be discussed considering all modified nanocomposite membranes.

## 2 Materials and methods

### 2.1 Materials

Cellulose acetate extra pure, (M.wt 50,000 with 29%–45% acetyl group), was purchased from LOBA Chem., India. Acetone ultrapure was obtained from sigma cosmetic. N, N-Dimethyl- formamide (DMF) was produced from Fisher chemical. Tetraethyl orthosilicate (TEOS) was purchased from Merck, Germany. Absolute ethanol (EtOH) purity 99.8% was supplied from Sigma Cosmetic, ammonia solution (33%) was supplied from Sigma Aldrich. Commercial Bentonite clay was provided from local market. Bovine serum albumin was produced from MP Biomedical.

### 2.2 Modification of fillers (TiO_2_ nanoparticles and Bentonite clay)

The chemical modification of TiO_2_ nanoparticles were carried out as follows: tetraethyl ortho-silicate (0.15 mL) hydrolyzed in a mixture of (100 mL ethanol and 1 mL ammonia) under stirring for 60 min at 50°C to form solution (A). TiO_2_ nanoparticles (0.3 g) were added to 6 mL of solution (A) and stirred for 2 h. at a speed of 400 rpm, stirring them for another 30 min with a speed of 15,000 rpm. The mixture was centrifuged at 6,000 rpm and washed several times with EtOH. The solid was dried in a vacuum oven at 80°C for 4 h. The Bentonite powder was physically modified by grinding, using a ball mill to form nano clay.

### 2.3 Preparation of nanocomposite membranes

Membranes were prepared by dissolving 8.5 g cellulose acetate (CA) in acetone (33.3 mL) and DMF (16.6 mL) under stirring at room temperature for 6 h. Different weight percentages from previously modified TiO_2_ NPs and nano-clay (0, 0.05, and 0.10 wt%) were added to the solutions with ultrasonication to prevent the agglomeration of nanomaterials after that the solutions kept serene for 12 h at room temperature to remove air bubbles. The mixtures were uniformly cast over glass plates using the automatic film applicator. The glass plates were immersed in distilled water at 25°C until membrane solidification in each case. Clay and titanium dioxide modified membranes with the abbreviations of CA/bent and CA/TiO_2_, respectively, were washed and kept in deionized water for 24 h before use.

### 2.4 Membranes characterization

Fourier transforms infrared (FTIR) spectra were recorded at room temperature for KBr discs in the range of 400–4,000 cm⁻^1^. The prepared samples were examined with a scanning electron microscope (JEOL, Model JSM 6360LA, Japan) to investigate the homogeneity and morphology of the samples. Before the investigation, the samples were coated with gold using a sputtering coater (model: S150B, Edwards High Vacuum Ltd., England). The quality of dispersion and also the existence of different fillers on the membrane surface were analyzed by EDX (Energy dispersion of X-ray JEOL). Thermo-gravimetric analysis (TGA) was studied at a heating rate of 20°C per minute under atmospheric nitrogen, while for differential scanning calorimetry (DSC) the heating rate is 10°C per minute. The hydrophilicity of the prepared UF membrane material is expressed in terms of contact angle which measures the wettability of the membrane. Contact angle measurement was executed using a Ram´e-Hart model. The tensile strength and percent elongation at break values of the membranes were determined at room temperature using the universal testing machine, Instron 3382 (100 KN), with a crosshead speed of 1 mm min^−1^ according to ASTM D871–96. Nanocomposite UF membranes were evaluated using cross-flow stainless-steel CF042, with a hydra pump. All details are presented in Supplementary Material.

## 3 Results and discussion

### 3.1 Fourier transform infrared (FT-IR)

**FIGURE 1:**
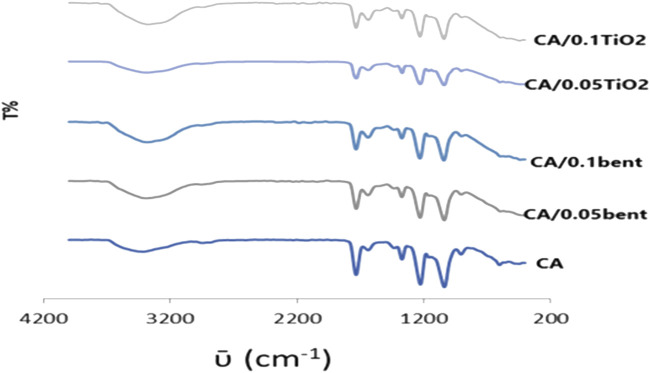
FTIR of prepared cellulose acetate nanocomposite membranes.

The FT-IR spectrum of the CA membrane displays bands at 1732vs, 1224vs., and 1035vs. cm^-1^due to **ν**_C=O_ acetyl, **ν**_C-O_ acetyl, and **ν**_c-o-c_ pyranose ring, respectively as shown in Figure 1. The ν_OH_ is traced as a broad medium band at 3,413 cm^−1^ while that due to **ν**-CH_2_- appeared as a weak band at 2,945 cm^−1^. The spectra display redshifts of **ν**_OH_ from 3,413 cm^−1^ in the CA membrane to 3,379, 3,385, 3,387, and 3,374 cm^−1^at CA/0.10bent, CA/0.05bent, CA/0.05TiO_2_, and CA/0.1TiO_2_ membranes, respectively. The bands at 400–600 cm^−1^ characteristic for Si-O and the weak bands at 1995–2,359 cm^−1^ are attributed to Ti–O stretching vibration and O–Ti–O lattice due to TiO_2-_polymer (George et al., 2014).

### 3.2 Scan electron microscope (SEM)

**FIGURE 2:**
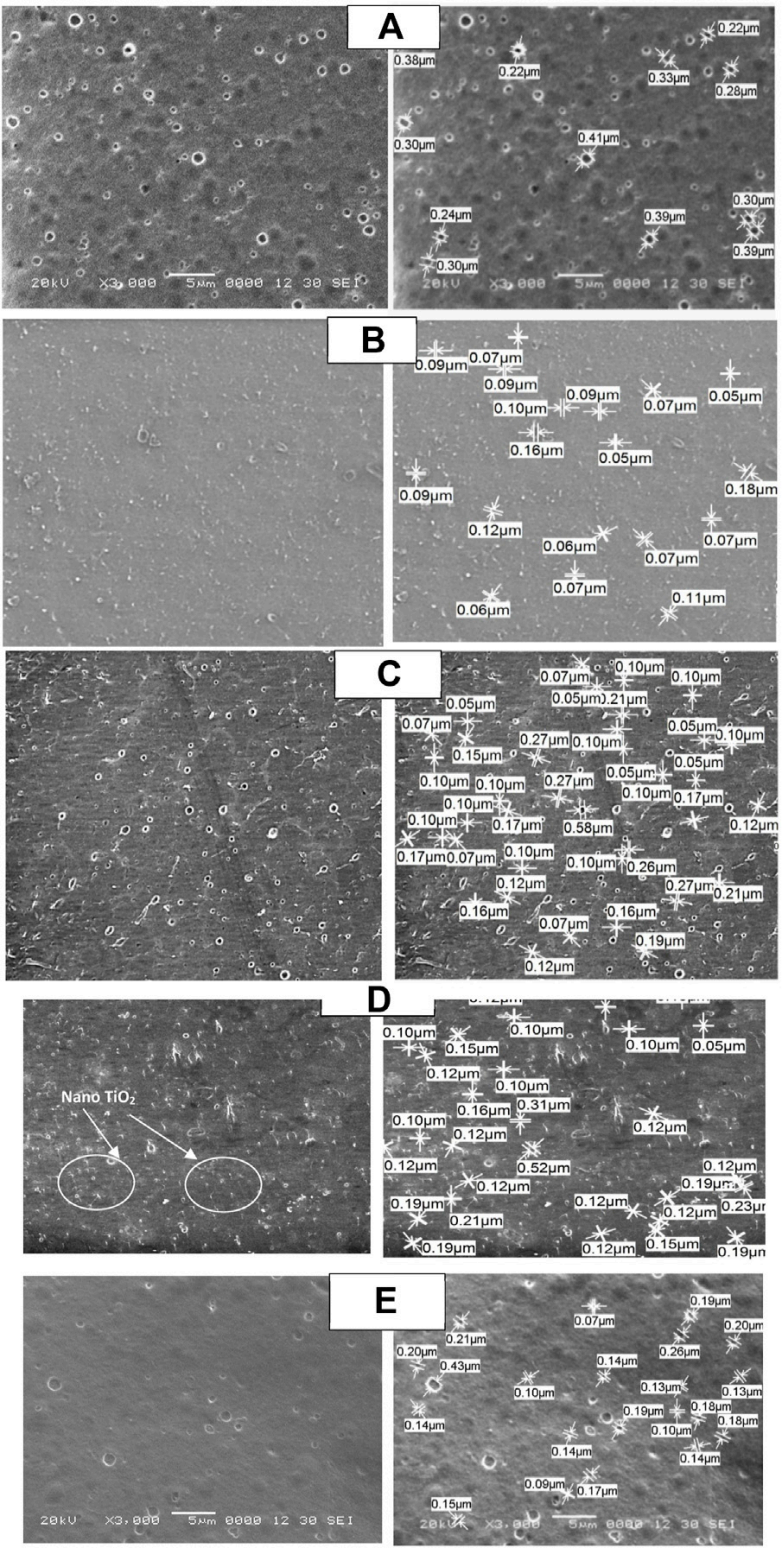
SEM micrographs of top surface (left) and pore diameter determination (right) of **(A)** neat CA, **(B)** CA/0.05 bent, **(C)** CA/0.1 bent, **(D)** CA/0.05 TiO_2_ and **(E)** CA/0.1 TiO_2_.

The SEM images of CA, CA/bent, and CA/TiO_2_ membranes are shown in Figure 2, indicating that the CA/bent and CA/TiO_2_ are more porous with smaller pore diameters than the CA membrane. The average pore diameter for CA is 0.31 µm and reduced to 0.1 µm after the addition of 0.05 wt% of bentonite clay while increasing the concentration of clay to 0.1 wt% of clay, some of the pore diameters increased to be 0.27 µm, and some decreased to be 0.05 µm with average pore diameter 0.11 µm. While for modified membranes with TiO2, the average pore diameter is lower than the average pore diameter of CA and higher than that of CA/bent. The average pore diameter of CA/0.05TiO_2_ and CA/0.1TiO_2_ are 0.16 µm and 0.2 µm, respectively. The best membrane is CA/0.05bent with the lowest average pore diameter and uniform pore size. . Furthermore, the images reflect the homogeneity and good dispersion of the clay and TiO_2_. The surface morphology, porosity, and homogeneousness of the dispersed Clay or TiO_2_ are necessary to enhance the membrane performance (Golobostanfard and Abdizadeh, 2013; Nivedita and Joseph, 2020).

### 3.3 Energy dispersive X-ray (EDX)

**FIGURE 3:**
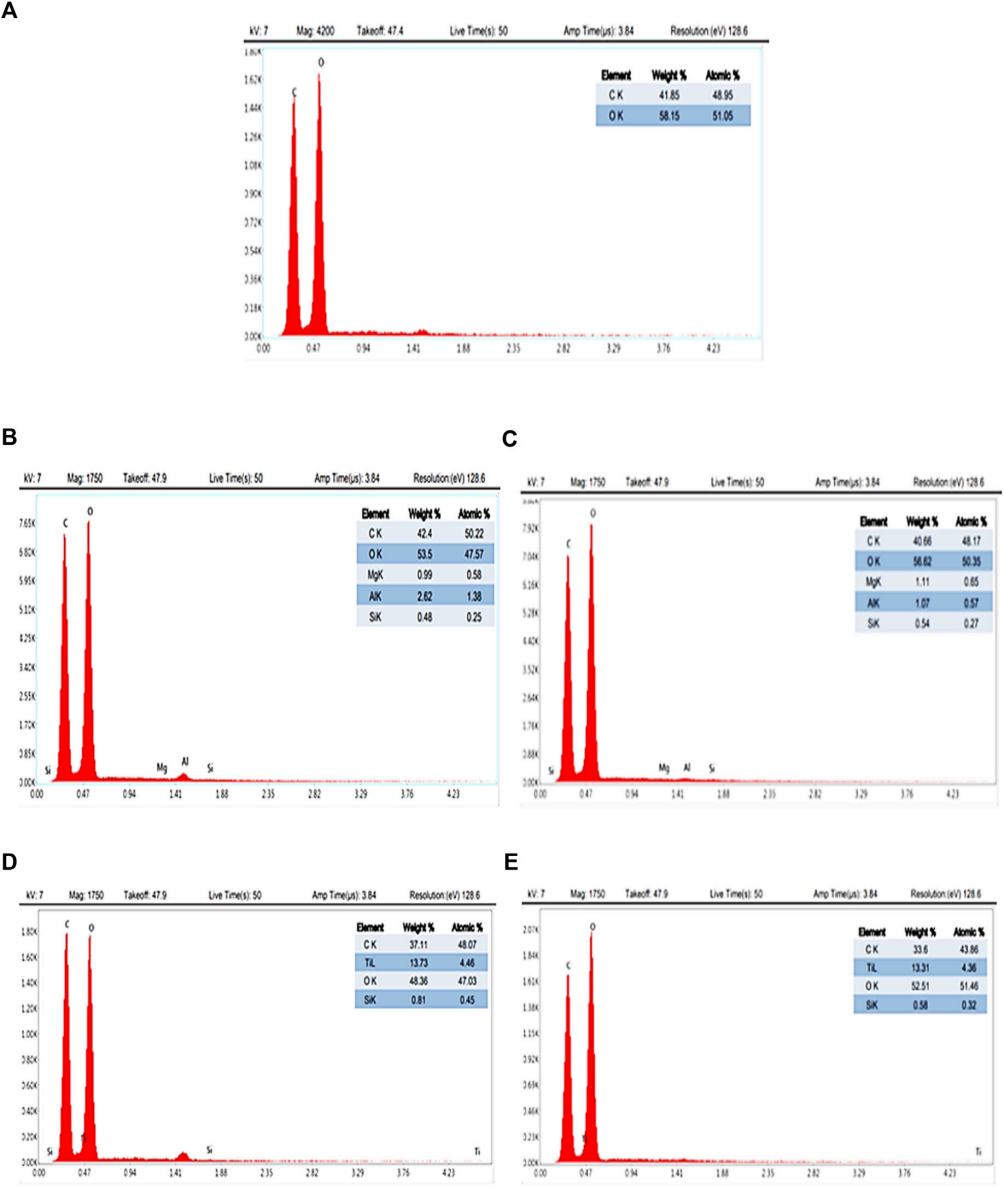
EDX for prepared nanocomposite membranes **(A)** neat CA, **(B)** CA/0.05 bent, **(C)** CA/0.1 bent, **(D)** CA/0.05 TiO_2_ and **(E)** CA/0.1 TiO_2_.

The EDX spectra of CA, CA/bent, and CA/TiO_2_ composite membranes are in Figure 3 (Joshi et al., 2015). The EDX spectra display only oxygen and carbon in the CA membrane, while the spectra of CA/0.05bent and CA/0.010bent exhibit peaks characteristic of Mg (0.99, 1.11%), Al (2.62, 1.07%), and Si (0.48% and 0.54%), respectively, with a reduction of C% and increase of O% compared to CA membrane. These data confirm the formation of CA/bent with the postulated clay contents and exhibit new peaks characteristic of Ti with weight percentages of 13.73% and 13.31% for CA/0.05TiO_2_ and CA/0.10TiO_2_, respectively. The peak characteristic of Ti, reduction of C%, and the increase of O% compared to pure CA confirm the combination of TiO_2_ with the membrane (Gebru and Das, 2016).

### 3.4 Thermal analysis

**FIGURE 4:**
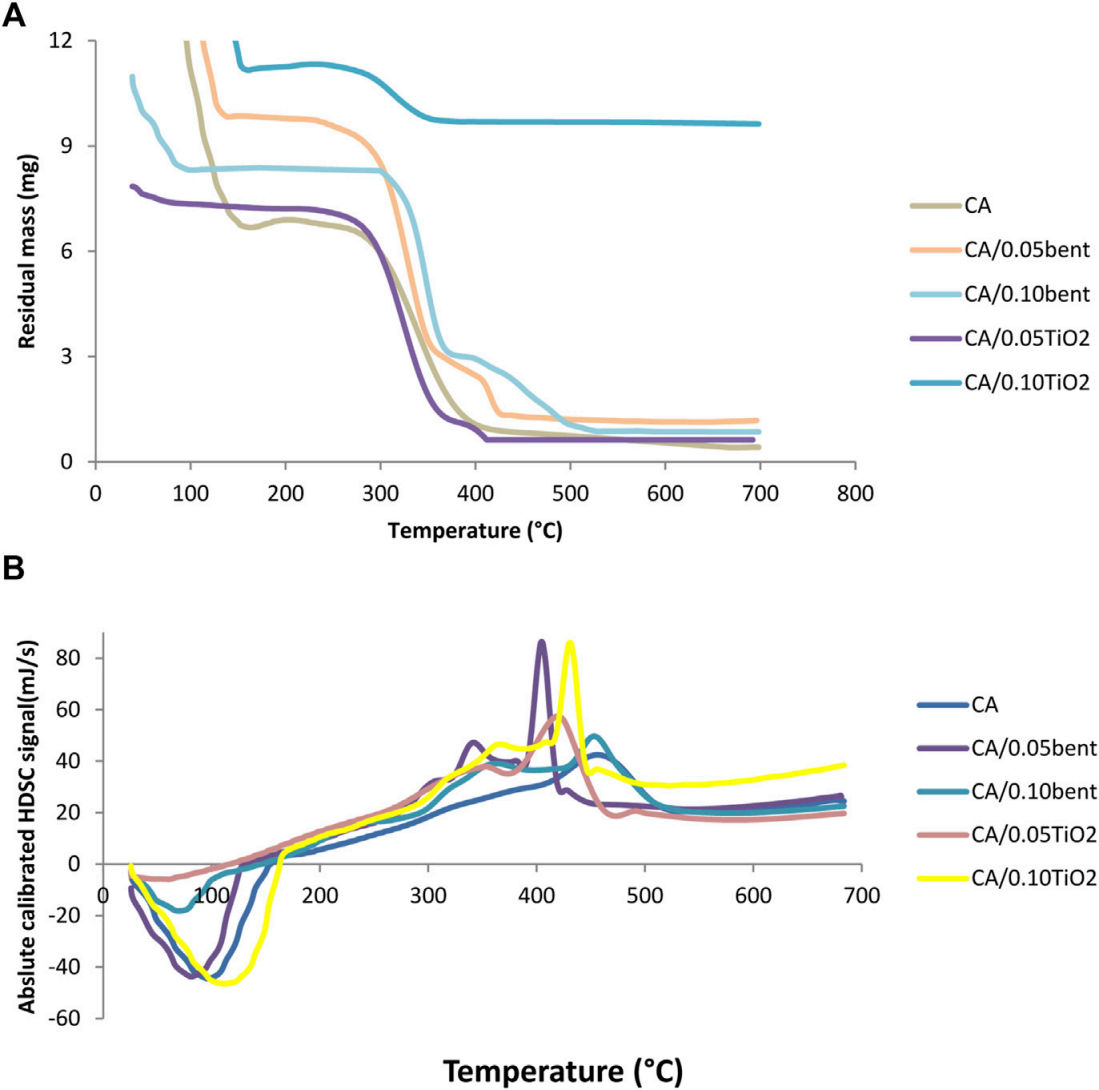
**(A)** TGA and **(B)** DSC for prepared cellulose acetate nanocomposite membranes.

Thermal analysis of CA, CA/bent, and CA/TiO_2_ composite membranes were performed using TGA and DSC and is shown in Figures 4A, B.

For the cellulose acetate membrane, the weight loss occurred through two individual steps at 298°C and 698°C. For modified membranes, a third step takes place at a temperature range of 410°C–441°C assumed for weight loss of inorganic components. The TGA results indicate that the thermal stability of each CA/bent and CA/TiO_2_ composite membrane is higher than the CA membrane. The DSC, Figure 4B, shows that the first peak is endothermic for all samples occurring between 73°C and 117°C, corresponding to the desorption of water molecules. The variance in the values of desorption peaks is attributed to the different water-holding abilities and polymer–water interaction. The second exothermic peak in the range of 334.3°C–380°C characteristic of melting of the membranes, confirming the formation of modified membranes. The third exothermic peak carries out at the highest temperature range, 400°C–690°C, because of breaking down intramolecular interaction and the decomposition of the polymer chain (Hong et al., 2020).

### 3.5 Membranes wettability

**FIGURE 5:**
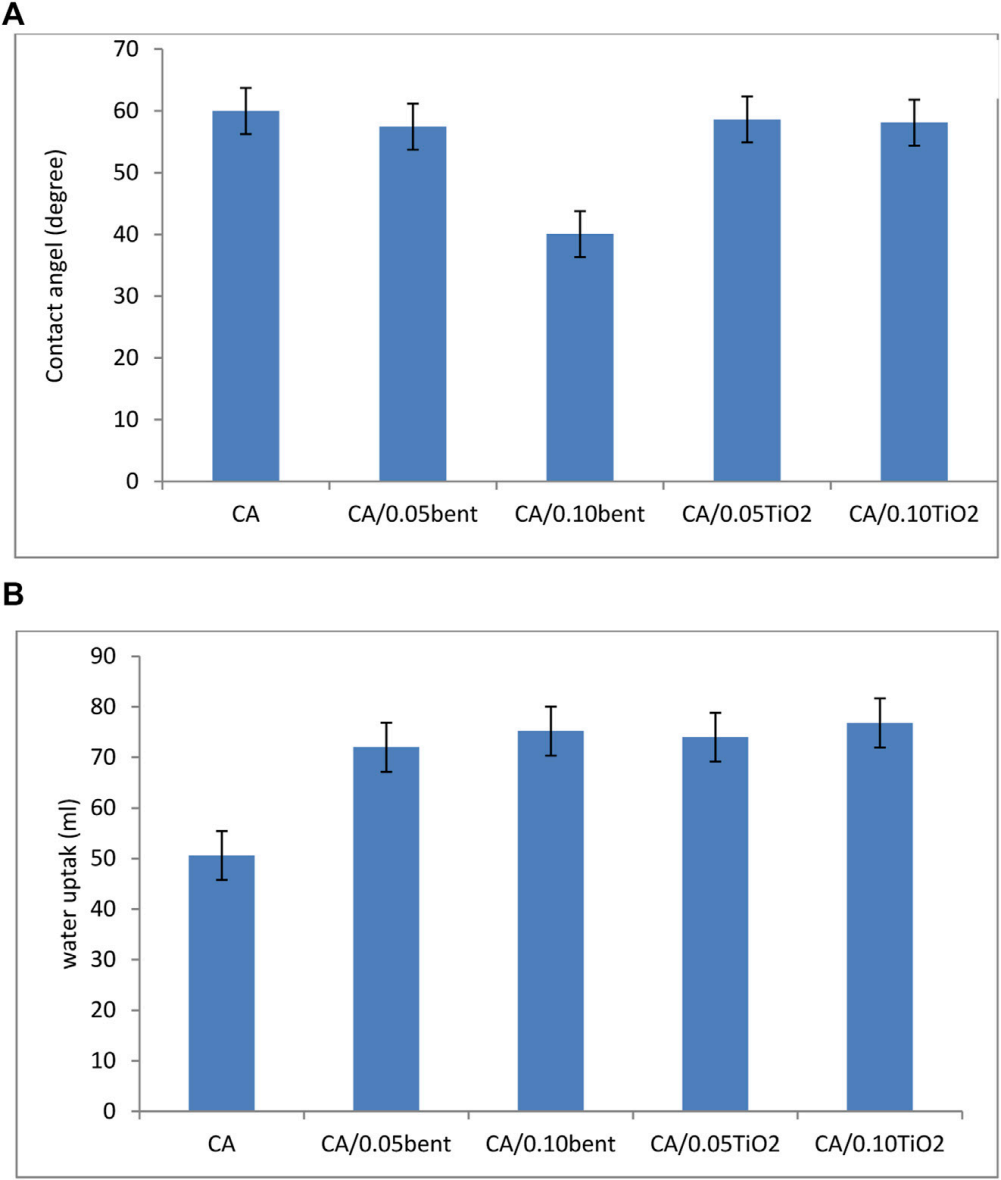
**(A)** Contact angel and **(B)** water uptake of prepared cellulose acetate nanocomposite membranes.

The surface wettability of CA and modified membranes were determined using contact angle measurement and presented in Figure 5A.

The contact angle of pure CA membrane and modified membranes with different clay and TiO_2_ loading is in Figure 5A. The results demonstrate that the contact angle of membranes decreased by adding the additives. The contact angle is inversely related to the hydrophilic property of the nanocomposite membranes. It means that a lower contact angle leads to a more hydrophilic membrane. Thus, when these super hydrophilic nanoparticles are inserted into a polymer matrix, they markedly enhance the hydrophilicity of the membrane (Shi et al., 2016). The data shows that the contact angle sequence is CA/0.10bent < CA/0.05bent < CA/0.10 TiO_2_ < CA/0.05 TiO2 < CA, the order of the hydrophilicity.

### 3.6 Membranes water uptake

The influence of the addition of different concentrations of bentonite clay or TiO_2_ to CA during membrane preparation on the water uptake is shown in Figure 5B.

Water Content measurements are assumed to be the mass difference between dried and swollen membranes (Duan et al., 2013). The data illustrated in Figure 5B resembles that the wettability of the membranes is in the order CA/0.10TiO_2_ < CA/0.05TiO_2_ < CA/0.10bent < CA/0.05bent < CA. The order of water uptake indicates that the CA/0.10TiO_2_ membrane exhibit the highest uptake referring to its high porosity and high oxygen concentration on its surface.

### 3.7 Ion exchange capacity (IEC)

The IEC is defined as milli-equivalents of ion-exchange groups per 1 g of the dry membrane using the titration method. The IEC is calculated using Eq. 1:

$$IEC=\frac{\left(V_{2}-V_{1}\right)a}{w}meq/g$$ (1)

Where V_1_ and V_2_: are volumes of NaOH required for complete neutralization of H_2_SO_4_ in the absence and presence of modified membrane, respectively, a: is the normality of NaOH, w: is the weight of membrane sample (Kalaiselvimary et al., 2017).

**FIGURE 6:**
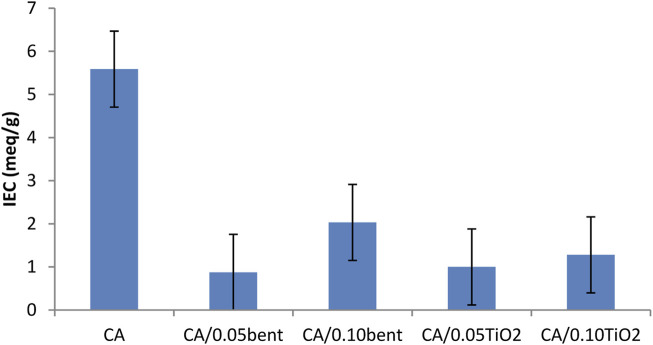
IEC of prepared cellulose acetate nanocomposite membranes.

The effect of the incorporation of clay or TiO_2_ on the IEC for the CA membrane is in Figure 6. The data exhibit a manifest decrease in IEC for all the modified membranes.

The decrease in IEC is due to i) a decrease in accessibility of ion exchange groups in the membrane matrix due to the presence of clay or TiO_2_ particles that can occupy the space around CA particles, ii) the higher-water uptake % of the modified membranes than the pure CA. These water molecules are strongly bonded to the active centers on the surface of the membranes and therefore decrease their exchange capacity.

### 3.8 Mechanical properties

The mechanical properties of CA, CA/bent, and CA/TiO_2_ composite membranes were set from the critical breaking point of broadening pieces, Table 1.

**TABLE 1:** Tensile strength and elongation% of prepared cellulose acetate nanocomposite membranes.

Sample	Tensile strength (N/cm^2^)	Elongation%
CA	10.3	4.09
CA/0.05bent	15.3	11.87
CA/0.1bent	10.5	4.8
CA/0.05TiO_2_	11.25	5.323
CA/0.1TiO_2_	10.6	5.067

One of the main reasons to incorporate inorganic particles into polymer matrices is to produce a membrane with improved mechanical properties (including tensile strength and the percent elongation at break). Tensile strength is the force essential for breaking the specimen or causing the complete separation of constituents in a linear direction. Elongation is the distance (in percent) the membrane will stretch from its original size to the point at which it breaks (Anuar and Zuraida, 2011). The tensile strength and elongation percentages of CA, CA/bent, and CA/TiO_2_ membranes are shown in Table 1. Generally, a large surface area of the nano-sized materials increases the interfacial interactions between the particles and the polymer matrix and consequently improves the mechanical properties of the formed nanocomposite membrane (Hassan et al., 2014).

The tensile strength of the membranes is in the order CA/0.05bent > CA/0.05TiO_2_ > CA/0.10 TiO_2_ > CA/0.10bent > CA. Therefore, the combination of nano-clay and Titania in the CA membrane enhances the tensile strength of the membrane. The results show that the highest tensile strength (15.3 N/Cm^2^) is for CA/0.05bent membrane attributed to the well-distributed modified clay without any aggregations in the cellulose acetate matrix can act as a defect inside the membrane as the filler percentage increase to 0.10 wt% (Mukherjee and De, 2014). The change in the elongation percentage of nanocomposite membranes is given in Table 1. The data shows that the elongation percentages are in the order of CA/0.05bent > CA/0.05TiO_2_ > CA/0.10 TiO_2_ > CA/0.10bent > CA. This order indicates that the elongation percentage of CA increases with the addition of the modified agents such as Clay and TiO_2_, Where the maximum elongation percentage is 11.78% for CA/0.05bent. The mechanical properties of modified CA/bent and CA/TiO_2_ membranes are higher than that of the pure CA membrane because the presence of NPs (TiO_2_ or Clay) acts as a bridge between different polymer molecules, making them close to each other (Mukherjee and De, 2014).

### 3.9 Effect of pressure on BSA rejection% and permeate flux

Instead of constant trans-membrane pressure (TMP) operation, membrane filtration is carried out under fixed flux conditions. In this case, the TMP becomes a dependent variable requiring an understanding of the relationship between permeation flux and TMP.

At low pressures, as the TMP increases, the flux increases linearly. At this point, the relationship is reversible, i.e., when the pressure decreases, the same original permeation fluxes can be re-established. At higher trans-membrane pressure, the particle concentration on the membrane surface and the permeation flux increase very slowly. At higher pressures, the permeation flux becomes pressure independent due to the consolidation of the particle deposit formed.

**FIGURE 7:**
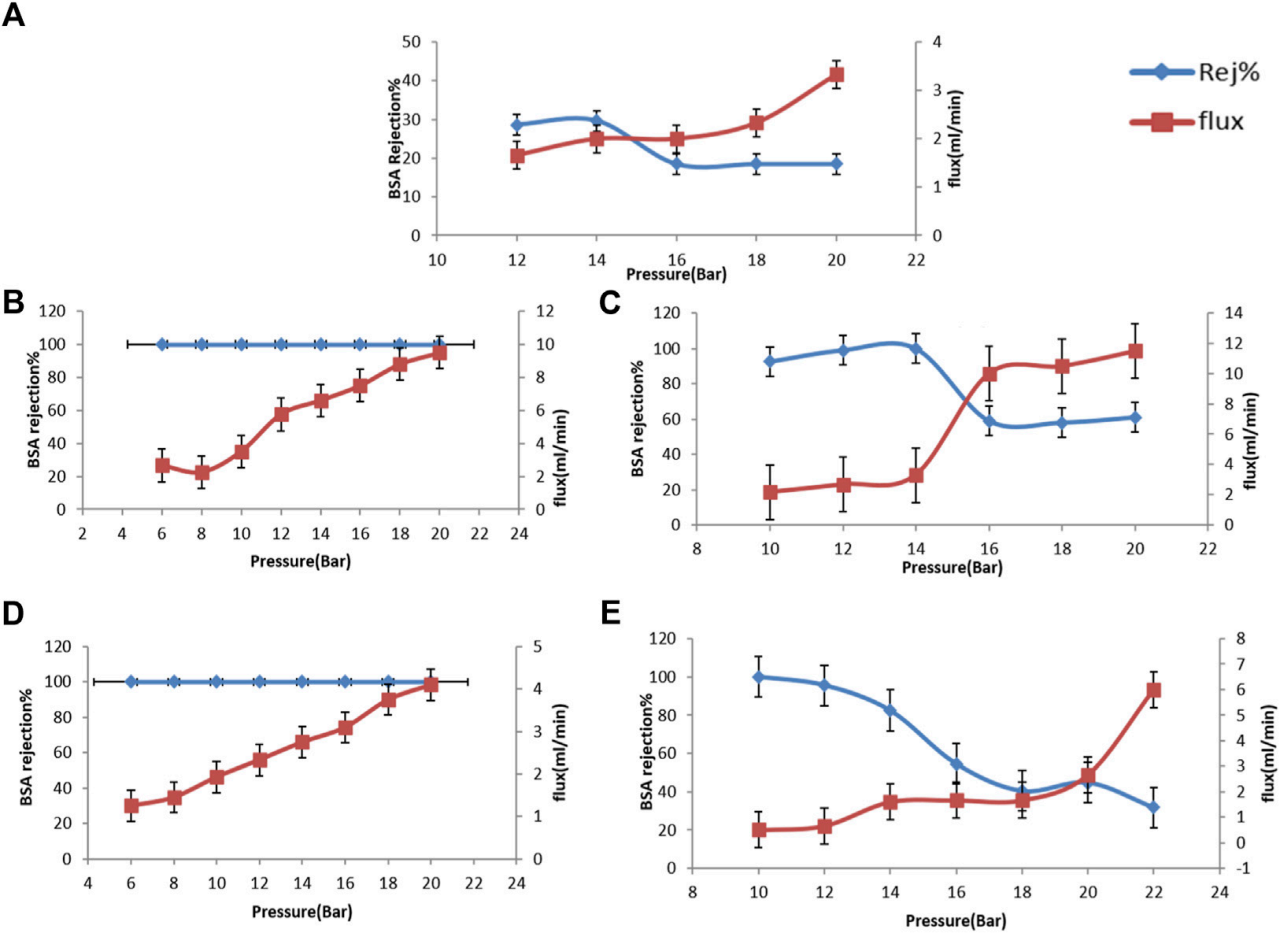
Rejection % and permeate flux of prepared cellulose acetate nanocomposite membranes, **(A)** neat CA, **(B)** CA/0.05 bent, **(C)** CA/0.1 bent, **(D)** CA/0.05 TiO_2_ and **(E)** CA/0.1 TiO_2_.

The feed pressure must be higher than the permeate pressure; otherwise, separation will not occur. Partial pressure difference will provide the driving force for the separation. When applying the permeate side at higher partial pressure, the driving force and permeate flux are diminutive. Besides that, if the porosity of the sublayer is too small, it can result in a high-pressure loss on the permeate side, increasing the tendency for capillary condensation to occur, confirming that the membrane selectivity is a significant factor (Csurka et al., 2022).The effect of variation of the pressure on bovine serum albumin (BSA) rejection percentage and water permeation flux through the CA, CA/bent and CA/TiO_2_ is explored in Figure 7, was measured at various applied pressure (12–20 bar).

The effect of variation of the pressure on bovine serum albumin (BSA) rejection% and water permeation flux through the CA, CA/bent, and CA/TiO_2_ explored in Figure 7A and measured at various applied pressure (12–20 bar). The data shows that upon increasing the pressure from 12 to 20 bar, the flux increased from 1.6 to 3.3 mL/min with decreasing in BSA rejection percentage from 30% to 18.5% at 20 bar. The permeate flux through CA/0.05bent (under applied pressure in the 6–20 bar range is illustrated in Figure 7B. While increasing the pressure from 6 to 20 bar, the flux increased from 2.6 to 9.5 mL/min with the stability of BSA rejection percentage around 100%. The data shown in Figure 7C shows that the permeate flux is increased from 2.16 to 11.5 mL/min upon increasing the pressure from 10 to 20 bar. While the BSA rejection percentage first increases to 100% at 14 bar and then decreases to 61% at 20 bar. The water permeation flux through CA/0.05TiO_2_ was increased from 1.25 to 4.1 mL/min with 100% BSA rejection upon increasing the applied pressure from 6–20 bar, as shown in Figure 7D. The permeate flux through the modified membrane CA/0.10TiO_2_ was measured at various applied pressure from (10 bar–22 bar). The flux increases from 0.5 to 6 ml/min with the decrease of BSA rejection percentage from 100% to 31.7% at 20 bar, as shown in Figure 7E. Figure (Yampolskii, 2012) shows that, as the applied pressure increases, the permeate flux for all prepared membranes increases with decreasing in the BSA rejection except for CA/0.05bent membrane. CA/0.05bent membrane exhibits the highest water flux (9.5 ml/min) with stable BSA rejection of 100%, showing the most efficient UF membrane. The membrane’s performance toward BSA removal from wastewater shows good enhancement due to the membrane’s moderate porosity having high hydrophilic nature and highest mechanical behavior.

## Conclusion

Titania nanoparticles and nano-clay were modified and incorporated into cellulose acetate to give nano-composite membranes. The membranes showed tremendous improvement in BSA rejection% from 18.5% for neat (CA) membrane to 100% for CA/0.05bent and CA/0.05TiO_2_ at the applied pressure of 20 bar. The most efficient membrane was the CA/0.05bent membrane that reached 100% BSA rejection for the applied pressure from (6 to 20 bar) with a higher permeates flux of 9.5 ml/min, and had the most mechanical stability and elongation%. All modified membranes were more hydrophilic than the neat cellulose acetate membrane according to contact angle measurements, water uptake, and IEC. The modified membranes had more thermal stability than the regular cellulose acetate membrane. The results showed that the modified membranes used effectively as ultrafiltration membranes.

## Data Availability

The original contributions presented in the study are included in the article/Supplementary Material, further inquiries can be directed to the corresponding authors.
