# Supplementary material for: Efficient removal of bovine serum albumin from water by cellulose acetate membranes modified with clay and titania nano particles
Source: Front Chem. 2023 Feb 1;11:1111558. doi: 10.3389/fchem.2023.1111558 (PMC9931067; doi:10.3389/fchem.2023.1111558)
Supplement: Supplementary file 1 [file DataSheet1.PDF]

2.4. Membranes performance test

Nanocomposite UF membranes were evaluated using cross-flow stainless-steel CF042, with a hydra-pump. All details are explained in supplementary file.. with maximum pressure (69 bars), pressure control valve and gauge through rejection line, membrane area (42 cm^2), flow meter F-550 (USA) to obtain constant flow 1 liter/min. Protein solutions (BSA) with a concentration of 1g/L were prepared using distilled water and filtered through each membrane individually. The BSA permeate concentration was estimated by UV-spectrophotometer at a wavelength of 280 nm. Prior to introducing BSA, membranes were flushed and compacted system with deionized water for at least 1 hour until a steady permeate flux was achieved. Once a steady permeate flux was obtained, each filtration run was operated under contact pressure mode. UF experiments were conducted with an initial feed concentration of BSA, the performance was evaluated in terms of flux and solute rejection of feed. The flux was calculated by measuring permeates penetrated through the membranes per area of the membrane per unit of time according to equation (1)(21) .

$$\text{Flux} = Q / A.\ \Delta t \qquad (1)$$

Where Q is the quantity of permeate (L), A is the effective membrane area (cm^2) Δt is the sampling time (h).

The solute rejection was achieved by determining the concentration of the feed solutions and the permeate concentration.

The rejection was calculated by the equation (2) (21):

$$\text{Rejection (\%)} = [(C_f - C_p)/C_f] \times 100 \qquad (2)$$

Where C_f is the concentration of the feed solution and C_p the permeate concentration.
